# Intron V, not intron I of human thrombopoietin, improves expression in the milk of transgenic mice regulated by goat beta-casein promoter

**DOI:** 10.1038/srep16051

**Published:** 2015-11-03

**Authors:** Yan Li, Hu Hao, Mingqian Zhou, Hongwei Zhou, Jianbin Ye, Lijun Ning, Yunshan Ning

**Affiliations:** 1 School of Biotechnology, Southern Medical University, North1838 Guangzhou Road, Guangzhou, 510515, PR. China; 2 Department of Pediatrics, the Six Affiliated Hospital of Sun Yatsen University, 26 Yun Cun Erheng Road, Guangzhou, 510655, PR. China; 3 School of Public Health and Tropical Disease, Southern Medical University, North1838 Guangzhou Road, Guangzhou, 510515, PR. China

## Abstract

Introns near 5′ end of genes generally enhance gene expression because of an enhancer /a promoter within their sequence or as intron-mediated enhancement. Surprisingly, our previous experiments found that the vector containing the last intron (intron V) of human thromobopoietin (hTPO) expressed higher hTPO in cos-1 cell than the vector containing intron I regulated by cytomegalovirus promoter. Moreover, regulated by 1.0 kb rat whey acidic protein promoter, hTPO expression was higher in transgenic mice generated by intron V-TPOcDNA than in transgenic mice generated by TPOcDNA and TPOgDNA. However, it is unknown whether the enhancement of hTPO expression by intron I is decreased by uAUG7 at 5′-UTR of hTPO *in vivo*. Currently, we constructed vectors regulated by stronger 6.5kb β-casein promoter, including pTPOGA (containing TPOcDNA), pTPOGB (containing TUR-TPOcDNA, TUR including exon1, intron I and non-coding exon2 of hTPO gene), pTPOGC (containing ΔTUR-TPOcDNA, nucleotides of TUR from uAUG7 to physiological AUG were deleted), pTPOGD (containing intron V-TPOcDNA) and pTPOGE (containing TPOgDNA), to evaluate the effect of intron I on hTPO expression and to further verify whether intron V enhances hTPO expression in the milk of transgenic mice. The results demonstrated that intron V, not intron I improved hTPO expression.

Human thrombopoietin (hTPO) is a major physiological regulator of steady-state megakaryocytopoiesis and platelet production. It is located on chromosome 3q27-q28 and encoded by a single gene consisting of 6 exons and 5 introns. Exons 1 and 2 contain 5′ untranslated region (5′-UTR) and the first 4 amino acids of the signal peptide, and the remainder of the secretary signal and the first 26 amino acids of the mature protein are encoded within exon 3^1^. An extra exon has been detected ahead of the first exon named as exon 0^1^. In physiological conditions, the expression of hTPO is very inefficient. It has been demonstrated that some special structures influence TPO expression level. Firstly, at the upstream of the transcription start site in TPO gene, there are no mammalian promoter elements (e.g., TATA, GC and CAAT boxes), but a cis-acting *ets* motif, ACTTCCG, which was discovered at 269 to 263 from the first initiation site for an optimal expression of the hTPO genome. It is well known that the interaction of ETS and TPO is the basic requirement for high expression of hTPO in hepatocytes. However, only one ETS element has been found in 5′ flanking sequence of hTPO gene. Single ETS is not able to form an efficient DNA-protein complex or trigger efficient transcription of hTPO^3^. Secondly, the translation of hTPO is strongly inhibited by the 7th uAUG (upstream translational start codons) that located in the 5′ UTR (exon1and non-coding exon2) of the hTPO gene. The 7th uAUG which is the nearest to physiological AUG exerts the strongest inhibition on TPO translation. The mutation was once made to remove the inhibition of uAUG and led to increasing TPO mRNA translation, causing elevated serum levels of TPO and the overproduction of platelets^4^. Interestingly, familial essential thrombocythaemia(ET), an autosomal-dominant disorder which results in increased level of TPO, was found to be caused by deletions in the 5′ UTR that removes or neutralizes upstream Open Reading Frames (uORF)^5^. Taken together, these findings demonstrate that hTPO expression is influenced by the structure of TPO gene so that the expression level of hTPO is always very low in physiological conditions. However, it is still uncertain whether there exist other specific elements in hTPO gene, which may enhance hTPO expression level when the body is stimulated by external factors (for example, chemotherapy, severe fever, blood purification).

Intron-containing genes are often transcribed more efficiently than non-intronic genes^6^. Based on the function of introns, the transcriptional regulation can be simply divided into two patterns: splicing-independent regulation and splicing-dependent regulation. In fact, the first category is capable of stimulating transcription because of the presence of an enhancer or a promoter element within their sequence. In the vast majority of cases, these regulatory elements are found within most of 5′- introns (first intron). In contrast, the transcription regulation of the second category requires a functional, splicing-competent intron among the body of the gene^7^. This direct effect of introns on gene transcription is often referred to intron-mediated enhancement (IME), which has been observed in diverse organisms including plants, insects, mice and human^8^,^9^,^10^,^11^. It has been shown that IME needs the presence of an intron near the 5′ end of the gene^12^. Contrary to the expectation, in our previous study, we found that the last intron (intron V) rather than the first intron significantly improved hTPO expression in cos-1 cell^13^ and the hTPO expression was more higher in the milk of transgenic mice carrying intron V-TPOcDNA than in transgenic mice carrying TPOcDNA and TPOgDNA under the control of 1.0 kb rat WAP promoter^14^. However, it is interesting but still unknown whether the effect of the first intron on enhancement of hTPO gene expression is weaken or neutralized by the presence of uAUGs at 5′-UTR of hTPO gene *in vivo*.

In 1994, two recombinant TPO, recombinant human TPO (rhTPO) and pegylated, recombinant human megakaryocyte growth and development factor (PEG-rhMGDF), began clinical development^15^. Unfortunately, the appearance of neutralizing antibodies against PEG-rhMGDF led to thrombocytopenia in some healthy volunteers. Although thrombocytopenia was never reported with rhTPO, the development of both recombinant proteins had been stopped in 2001. Because of this unexpected side-effect, a new generation of TPO receptor agonists, romiplostim and eltrombopag, has been developed^16^,^17^. The application of mammary gland bioreactors of transgenic animal to produce pharmaceutical proteins with high-level expression and the appropriate post-translational modifications have become an attractive method. For example, proteins such as α1-antitrypsin^18^, tissue plasminogen activator^19^ and protein C^20^ have been expressed in the milk of transgenic animals. In order to obtain large and natural structure of recombinant hTPO, which needs complex post-translational modifications like high glycosylation and avoiding the production of antibodies against rhTPO in clinical practice, transgenic animal bioreactors could become an attractive alternative of preparation of recombinant hTPO. Based on our previous study, under the control of the 1.0 kb WAP promoter, the highest TPO levels (2.3 ng/ml) were found in transgenic mice carrying intron V-TPOcDNA, which were 5 ∼ 10 times higher than in the transgenic mice carrying TPOcDNA and transgenic mice carrying TPOgDNA, respectively^14^. Therefore, we speculated that the last intron probably enhance hTPO expression in the milk of transgenic animals, especially large transgenic animals, which would be benefit for producing abundant recombinant hTPO with natural structure in therapeutic application.

In current study, the stronger promoter of 6.5 kb **β**-casein was used to construct 5 mammary gland-specific expression vectors containing different exon-intron configurations of hTPO gene to evaluate the effect of the first intron on hTPO expression in cell and transgenic mice when the 7th AUG was deleted and further verify whether the last intron of hTPO gene could enhances the expression of hTPO in transgenic mice.

## Results

### Construction of hTPO mammary gland-specific expression vectors

**Figure 1:**
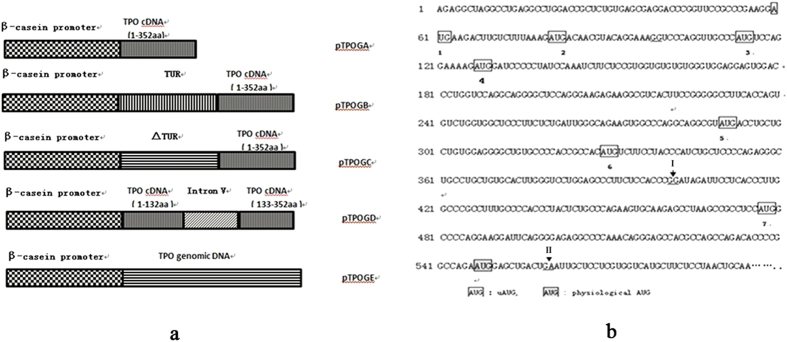
Constructions for hTPO mammary gland specific expression vectors and description for uAUG position in the 5′UTR of hTPO gene. (**a**) Scheme for five hTPO mammary gland specific expression vectors. 1.0 kb hTPOcDNA was cloned into pCβ-casein vector to construct recombinant expression vector pTPOGA. TUR- TPOcDNA and ΔTUR- TPOcDNA was respectively cloned into pCβ-casein vector to construct recombinant expression vector pTPOGB and pTPOGC. 1.3 kb TPO intron V-TPO cDNA was cloned into pCβ-casein vector to construct recombinant expression vector pTPOGD. 6.2 kb TPO gDNA was cloned into pCβ-casein vector to construct recombinant expression vector pTPOGE. TUR: containing exon I, intron I and non-coding exon2 of hTPO gene; ΔTUR: All nucleic acids were deleted from uAUG7 to physiological AUG in TUR. (**b**) Position of uAUGs in the TPO 5′-UTR. The full-length mRNA sequence of the TPO 5′-UTR is shown. Upstream AUG codons are boxed and numbered in the order as they appear. Triangles and Roman numerals mark the locations of introns.

To evaluate the intron effect on hTPO expression in mice, the 6.5kb goat β- casein promoter was used to construct 5 mammary specific expression vectors, which respectively contained TPOcDNA(pTPOGA), TUR-TPOcDNA(pTPOGB), ΔTUR-TPOcDNA(pTPOGC), intron V-TPOcDNA (pTPOGD) and TPOgDNA (pTPOGE) shown in Fig. 1a. TUR was constructed to contain exon1, intron I and non-coding exon2 of hTPO gene, more importantly, containing uAUG5 ∼ uAUG7. In order to eliminate the effect of uAUG7 on intron I influence on the expression of hTPO gene, ΔTUR was deleted from uAUG7 to physiological AUG to at TUR, but still contained the correct splicing sequence of introns and exons (Fig. 1b). These recombinant vectors were confirmed by restriction enzyme digestion and gene sequencing (data not shown).

### Expression of hTPO in the supernatant of HC-11 cells

**Figure 2:**
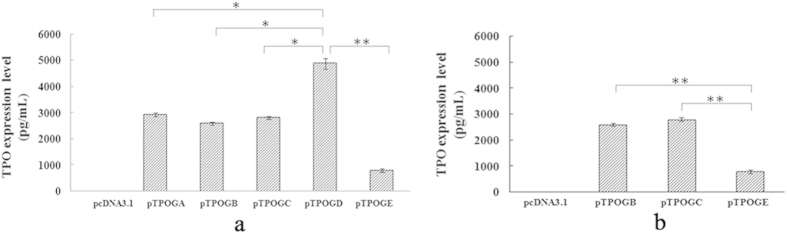
hTPO expression level of 5 mammary gland-specific expression vectors in HC-11 cells. Five mammary gland-specific expression vectors were introduced into cultured HC-11 cells for transient expression for 48h after DOTAP liposome-mediated transfection. The supernatants of cells were analyzed by using Human TPO Immunoassay Kit. Each column represents the mean  ±  standard deviation of 3 independent experiments. HC-11 cell without transfection was used as a blank control with the empty vector as a negative control. Five columns were compared by one-way ANOVA using SPSS13.0 program and the confidence interval is 95%. Significance was defined by p value of < 0.05. *p < 0.05, **p < 0.01. (**a**) The effect of intron V on hTPO expression; (**b**) The effect of uAUG7 and intron I on hTPO expression.

To optimize the transfection condition, HC-11 cells were firstly transfected with pcDNA3.1 GFP and the transfection efficiency was 56% at 48 h after transfection. Then HC-11 cells were respectively transfected with these 5 mammary gland-specific expression vectors for 48 h by DOTAP liposomes. pCDNA3.1 zeo(-) was used as control. The supernatants were collected to detect the expression level of hTPO by highly sensitive sandwich ELISA and all 5 specific mammary gland-specific vectors were all expressed in HC-11 cells (shown in Fig. 2). The expression level of hTPO was the highest in pTPOGD (4900 pg/mL) and the lowest in pTPOGE (797 pg/mL). Moreover, the hTPO expression was statistically significant higher in HC-11 cells transfected with pTPOGD than in those transfected with other vectors (Fig. 2a). Although pTPOGB and pTPOGC contained intron I and uAUG7 at non-coding exon2 of hTPO gene, uAUG7 was deleted in the latter. There was no statistical difference of hTPO expression level between pTPOGB and pTPOGC (Fig. 2b). In addition, pTPOGB and pTPOGE contained the same uAUG7, but the expression level of hTPO from the pTPOGB was far higher than that from the pTPOGE (Fig. 2b).

### PCR detection for transgenic mice

To preliminary screen positive transgenic mice, PCR was performed on the tail genomic DNA of mice. The primers P1 and P4 were used to amplify the tail genomic DNA of 22 F0 mice generated by pTPOGA vector. A PCR product of 620 bp was observed in 5 transgenic mice and not detected in non-transgenic mice (shown in Supplementary Fig. S1-a online). The primers P1 and P4 were used to amplify the tail genomic DNA of 51 F0 mice generated by pTPOGB vector. A PCR product of 620 bp was observed in 3 transgenic mice and not detected in non-transgenic mice, as shown in Supplementary Fig. S1-b online. The primers P1 and P4 were used to amplify the tail genomic DNA of 47 F0 mice generated by pTPOGC vector. A PCR product of 620 bp was observed in 4 transgenic mice and not detected in non-transgenic mice (shown in Supplementary Fig. S1-c online). The primers P2 and P3 were used to amplify the tail genomic DNA of 69 F0 mice generated by pTPOGD vector. A PCR product of 610 bp was observed in 4 transgenic mice and not detected in non-transgenic mice (shown in Supplementary Fig. S1-d online). The primers P2 and P3 were used to amplify the tail genomic DNA of 61 F0 mice generated by pTPOGE vector. A PCR product of 610 bp was observed in 2 transgenic mice and not detected in non-transgenic mice (shown in Supplementary in Fig. S1-e online).

### Detection for hTPO expression in the milk of transgenic mice

To detect the concentration of hTPO in the milk of transgenic mice, a high sensitive hTPO sandwich ELISA was used. The collected milk was diluted 1:5 with pyrogen-free water and lipids were removed by centrifugation. Then the milk samples were analyzed together with milk samples of normal mice, which served as the negative control. The mean concentration of hTPO for 7 transgenic mice was listed in Supplementary Table S1 online. Transgenic mice # 38 carrying intron V-TPOcDNA expressed the highest level of hTPO (146.00 ng/mL) and transgenic mice # 16 carrying TPOgDNA expressed the lowest level of hTPO (0.49 ng/mL). Although carrying ΔTUR-TPOcDNA, the expression of hTPO in the milk of transgenic mices #10(2 ng/ml) and #46(1.1 ng/ml) is similar to the transgenic mices #50(1.22 ng/ml) carrying TUR-TPOcDNA.

**Figure 3:**
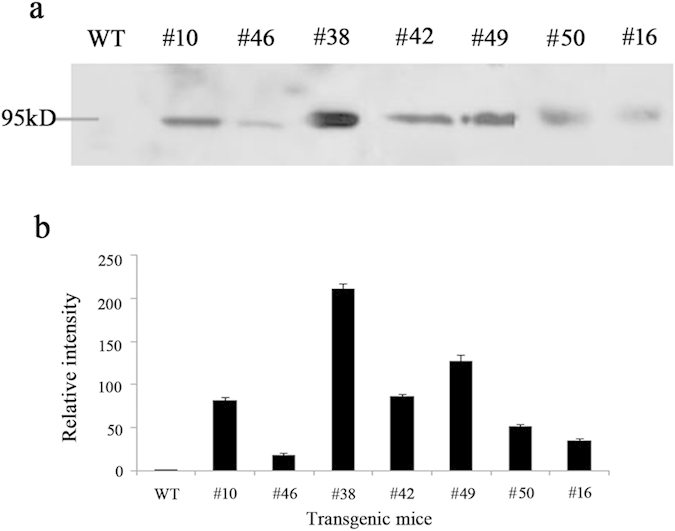
Identification of hTPO expression in the milk of transgenic mice by western blot. Milk samples were diluted 1:2 in PBS and 20 μl of each sample was loaded under reducing conditions onto a SDS-PAGE gel (10%). Subsequent western blot was performed according to standard protocols. An HRP-conjugated anti-hTPO antibody from the kit diluted 1:300 was used. (**a**) Western blot. Lane 1: milk samples from non-transgenic mice; lane 2–8: milk samples from seven transgenic mice lines. (**b**) Band relative intensity of hTPO expression was quantified by Gel-Pro Analyzer 4.0 software.

Furthermore, the expression of hTPO in the milk of transgenic mice was detected by western blot. Milk was collected from 7 female transgenic mice at day 8–12 of lactation, 1:2 diluted in PBS and 20 μl was loaded. As a result, specific hybridization bands having an apparent molecular mass of about 95kD were detected by anti-hTPO antibody, while no corresponding signal was detected in non-transgenic milk (shown in Fig. 3). Statistical analysis was performed after the band relative intensity was quantified by by Gel-Pro Analyzer 4.0 software (shown in Supplementary Fig. S2 online). These results showed that the recombinant hTPO was expressed in the milk of transgenic mice and the expression level was higher in transgenic mice generated by pTPOGD than others.

### hTPO expression in the mammary gland of lactating goat

**Figure 4:**
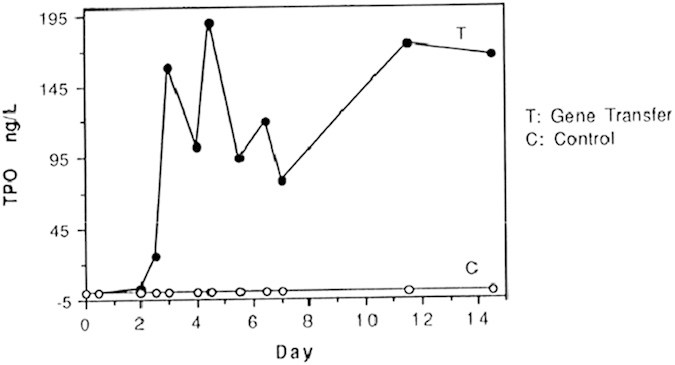
hTPO expression in the milk of goat after the transfer of pTPOGD vector. pTPOGD and pCDNA3.1 zeo(-) were purified by EndoFree Plasmid Maxi Purification Kit, embedded by liposome and then injected into two groups of goat mammary gland. The milk was collected after 12 h for continual 14 days and hTPO expression level was detected.

In cell and transgenic mice experiments, pTPOGD vector containing intron V-TPOcDNA induced the highest hTPO expression level. To further confirm this result, pTPOGD mixed lipofectin was injected into the base of mammary gland of lactating goats. The hTPO could be detected for 2 weeks in the milk of goat and the highest concentration attained to about 195ng/mL (shown in Fig. 4).

### hTPO mRNA expression in HC-11 cells transfected with pTPOGA and pTPOGD

To further explore whether intron V-mediated enhancement of hTPO expression was related to increasing hTPO mRNA level, pTPOGD (a natural intron V-containing gene) and pTPOGA (an intronless gene) were respectively transfected into HC-11 cells for 48 h and quantitative RT-PCR was performed to compare hTPO mRNA expression. The resulted showed that intron V-containing constructs (pTPOGD) yielded significantly more mRNA than the non-intron control(pTPOGA) (shown in Supplementary Fig. S3 online).

### hTPO mRNA expression in HC-11 cells transfected with pTPOGD and pTPO^△^GD

**Figure 5:**
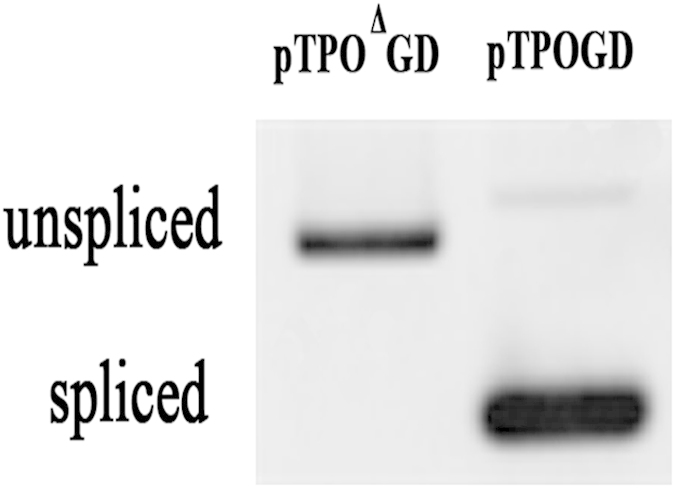
Splicing as a requirement for intron V-mediated enhancement of hTPO transcription. RT-PCR was performed to compare hTPO mRNA expression in HC-11 cells transfected with expression vector pTPO^Δ^GD and pTPOGD, respectively. Intron V with a mutated 5′ splice site was not removed from hTPO pre-mRNA as indicated by the position of RT-PCR product.

To further determine whether intron V-mediated enhancement of hTPO transcription was due to the splicing function of intron V, pTPO^△^GD containing the 5′ splice site of hTPO intron V mutated from GT to CA was constructed and could not be spliced out of hTPO precursor mRNA and a longer transcript was produced (shown in Fig. 5). Compared with pTPOGD, quantitative RT-PCR revealed that intron V-mediated transcriptional stimulation of hTPO was decreased by approximately 30 ∼ 40% under pTPO^△^GD transfection (shown in Supplementary Fig. S4 online). Thus, the intron V-mediated enhancement in hTPO mRNA depends on its splicing function.

## Discussion

The liver is the major site of hTPO biosynthesis and the normal serum concentration of hTPO is very low ranging between 0.5 ∼ 2 pmol/L^4^,^21^,^22^,^23^. It has been proved that the hTPO expression is inhibited through special structures of hTPO gene, such as having multiple transcription initiation sites, lacking a putative TATA box near the initiation sites and containing only 1 ETS element. Another importance discovery is that the translation of hTPO mRNA is almost inhibited by the 7th uAUG codon in the 5′-UTR^4^. However, it is still uncertain whether there exist other specific elements in hTPO gene, which may enhance TPO expression level when the body is stimulated by external factors, such as chemotherapy, severe fever and blood purification.

Introns are important regulators that can facilitate gene rearrangement, increase protein diversity and control expression at many different levels^24^. Removing the introns from a gene often significantly reduces its expression level^8^. The expression level of intronless-transgenes in mammalian cells is often 10 ∼ 100 times lower than their intron-containing counterparts^25^,^26^. Taken together, the expression level of genomic DNA is, much higher than that of cDNA in general. More and more studies have demonstrated that introns, especially intron I, significantly elevated gene expression level^27^,^28^,^29^. Surprisingly, in this study, pTPOGD (carrying the last intron) obtained the hTPO expression level of 4900 pg/mL in HC-11 cell, which was about 1.75 times higher than pTPOGB/pTPOGC (carrying intron I) and was 6 times higher than pTPOGE (carrying all introns) (Fig. 2a). Meanwhile, the hTPO expression attained to the highest level of 146 ng/ml in the milk of transgenic mice generated by intron V-TPOcDNA, which was about 120 times higher than in transgenic mice containing TUR –TPOcDNA (Supplementary Table S1 online). Because of TUR containing intron I as well as uAUG7, whether the presence of uAUG7 abates the effect of introns I on the improvement of TPO gene expression remains to be figured out. For this, ΔTUR was constructed in which uAUG7 was deleted in TUR (Fig. 1). However, the hTPO expression level was no obvious difference between the deletion and wild type in HC-11 cells and in the milk of transgenic mice (Fig. 2b and Supplementary Table S1 online). Moreover, the hTPO expression in the milk of transgenic mice containing ΔTUR -TPOcDNA is much more lower than in the milk of transgenic mice containing intron V-TPOcDNA. Taken together, these findings and our previous results demonstrated that the last intron, not the first, significantly increased expression level of hTPO in cell experiments and transgenic mice, and probably played the regulatory role in hTPO expression. Therefore, it is well worth exploring the mechanism that the last intron improves the hTPO expression level in normal and disease conditions. Another interesting finding is that pTPOGB and pTPOGE contained the same uAUG7(Fig. 1), but in HC-11 cell, the hTPO expression level in pTPOGB was far more higher than in pTPOGE (Fig. 2). These results implied that there are other elements in the hTPO genome to inhibit gene expression.

At present, many introns that affect gene expression increase mRNA accumulation through an unknown mechanism, may refer to enhancing transcription or the mRNA stability^30^, increasing nucleocytoplasmic transport of mRNA and translational efficiency^31^,^32^. In the vast majority of cases, only a promoter-proximal intron can enhance gene transcription^26^. In present study, the results showed that hTPO mRNA level in the presence of intron V was significantly more than in the absence of intron V. Further studies demonstrated that intron V-mediated enhancement of hTPO at mRNA level depended on its splicing function. These findings are contrary to the established view that only a promoter-proximal intron can enhance gene expression. The mechanism in which a intron located away from the promoter enhances mRNA level of hTPO will be explored in further research.

It is a demand of bioengineering to produce pharmaceutical proteins in the mammary gland of transgenic animals. A few human cytokines such as IL-2^33^ and GM-CSF^34^ have been expressed in the milk of transgenic animals, with large expression levels at around several milligrams per milliliter. However, there are still many obstacles in the development of such technology. One is how to improve the quantity of target gene expression. Several studies have shown that genomic constructs were expressed more efficiently in transgenic animal than other similar constructs except for the lack of introns^10^,^35^. Different from α1-antitrypsin, tissue plasminogen activator and protein C, hTPO gene contains special structures, such as being lack of TATA box, only having one ETS binding site, containing 7 uAUGs and therefore it is not feasible to construct high TPO expression vectors in the mammary gland of transgenic animal using TPO genomic structure. In our previous study, intron V enhanced hTPO expression in the milk of transgenic mice under the control of 1.0 kb rat WAP promoter^14^, which paved the way for producing high level of rhTPO in the milk of transgenic animal. Therefore, in present study, 6.5 kb β-casein promoter, which was stronger than WAP promoter, was used to construct 5 mammary gland-specific expression vectors to further verify the role of intron V in enhancing hTPO expression. The results showed that rhTPO expression attained to the highest level of 146 ng/ml in the milk of transgenic mice carrying intron V-TPOcDNA, which was about 18 times and 290 times higher than transgenic mice carrying TPOcDNA and TPOgDNA. The tendency was similar with previous study^14^.

In current study, seven F0 female transgenic mice were generated and detected hTPO expression level in the milk by ELISA and Western blot without detecting copy number. Accumulating evidence demonstrated that no direct association was observed between the copy number and the amount of recombinant proteins produced by transgenic mice^36^,^37^,^38^,^39^. Burkov *et al*., showed that despite equal copy numbers of the transgene in offspring of #9 and #11 founders, secretion of hGM-CSF in these animals differed. Moreover, offspring from #3, having 200 copies of the pGoatcasGMCSF transgene, did not show high level of hGM-CSF secretion^37^. In this study, there were two female transgenic mice carrying the same construct (ΔTUR -TPOcDNA) and hTPO expression level in milk was 1.1 ± 0.63 ng/mL and 2.00 ± 0.48 ng/mL without significant difference. There were also two female transgenic mice carrying the same construct (intron V-TPOcDNA) and hTPO expression level in milk was 122.80 ± 38.49 ng/mL and 146.00 ± 26.55 ng/mL without significant difference. Other constructs only generated one female transgenic mouse. Among them, the hTPO expression level was the highest in transgenic mice carrying intron V-TPOcDNA. Taken together, intron V significantly improved hTPO expression consistent with our previous research in which the last intron also enhanced hTPO expression in the milk of transgenic mice under the control of 1.0 kb rat WAP promoter^14^. Further, the expression level of the former was about more 63 times than that of the latter.

In order to investigate whether the last intron V also improve hTPO expression in large animals under the control of 6.5 kb β-casein promoter, the vector pTPOGD harboring intron V was embedded in liposome and then injected to the mammary gland of 3 lactating goat. The hTPO was continually detected in the milk of one goat for 14 days and the measured highest concentration was 190 ng/mL (Fig. 4). Because of β-casein promoter being a strong mammary gland tissue-specific regulatory sequence, it was possible that hTPO could obtain high level expression in the milk of transgenic goat.

Clinical studies have shown that rhTPO and PEG-rhMGDF were potent stimulators of platelet production and increased platelet with immune thrombocytopenia, chemotherapy-induced thrombocytopenia and myelodysplastic syndromes^40^. However, PEG-rhMGDF was a truncated non-glycosylated protein expressed in *Escherichia coli* and coupled to polyethylene glycol to improve stability. In some healthy volunteers who received this agent, neutralizing antibody against PEG-rhMGDF was detected and these antibodies neutralized its activity but also cross-reacted with and neutralized endogenous thrombopoietin to produce a paradoxical thrombocytopenia. The production of neutralizing antibody may be associated with molecular structure and glycosylation which are different from the natural molecule^17^. Moreover, amino acid sequence in rhTPO expressed in CHO cells was identical to that of endogenous thrombopoietin with a slightly different glycosylation pattern. Although there was no reported thrombocytopenia because of using rhTPO, the development of both recombinant proteins had stopped in 2001^16^,^17^. At present, rhTPO is approved (http://www.sfda.gov.cn/WS01/CL0001/) only in China, but the expression level is low. The above mentioned problem can get a certain degree of solution by the application of transgenic animal bioreactors with the high-level expression and the appropriate post-translational modifications^41^. Our study demonstrated that last intron significantly increased hTPO expression level in cell experiments and transgenic mice, which paved the way for obtaining high expression of hTPO in transgenic animal bioreactors. In subsequent experiments, we will use much stronger lactoprotein promoters to express high amounts of hTPO in the milk of large transgenic animals. Therefore, we anticipate that the production of functionally active hTPO in the milk of transgenic animals will be an efficient alternative to obtain large quantities of therapeutic proteins for the treatment of various diseases.

In conclusion, our present study further confirmed that the last intron, not the first intron, significantly increased hTPO expression level. This observation will provide a better understanding of the mechanism that the last intron may play a role during hTPO gene expression regulation. Furthermore, developing a mammary gland-specific expression vector that could be used to produce large amounts of hTPO in milk will be served as a platform for producing other potential therapeutic drug proteins efficiently and at low cost.

## Methods

### Construction of TPO mammary gland specific expression vectors

See supplementary methods

### ELISA for hTPO expression in supernatant of cell culture

HC-11 cells were cultured in hormone-induced medium (RPMI 1640, 10% fetal bovine serum, 10 ng/ml epidermal growth factor, 5 μg/ml human insulin). When cells grew to 60–70% confluent monolayers, the medium was poured out. After being washed twice with medium, the cells were then continually cultured for 4 days in culture medium (2% fetal bovine serum, 10 ng/ml epidermal growth factor, 5 μg/ml human insulin). Then, hormones (5 μg/ml human insulin, 5 μg/ml bovine prolactin, 0.1 μmol/L dexamethasone) were added to induce cell growth.

To optimize transfection conditions, cells were firstly transfected with pcDNA3.1-GFP (Invitrogen) to verify the transfection method. Transient transfection was carried out according to the protocol of DOTAP Liposome Transfection Reagent (Roche Applied Science, Germany) and green fluorescent cells were counted under a fluorescence microscope to determine the transfection efficiency. After that, the target vectors were transfected respectively. Briefly, 5 μg DNA was diluted with HBS buffer to a final volume of 50 μl and DOTAP was mixed with HBS buffer to a final volume of 100 μl. Then, the DNA solution was transferred to the DOTAP solution and the transfection mixture was carefully mixed by gently pipetting, added to the medium (2 ml RPMI 1640 supplemented with 10% fetal serum), and incubated for 15 min at 20 °C. After that, the mixture was added to the cells which were continually cultured for 9 h. The medium was removed and the cells were washed 3 times with RPMI 1640. Then hormones (5 μg/ml human insulin, 5 μg/ml bovine prolactin, 0.1 μmol/l dexamethasone) were added to the cells for 24, 48, and 72 h, respectively. The supernatants were collected by centrifugation at 4,000 *g* for 10 min and stored at −20 °C for measurement. A specific Human TPO Immunoassay kit (R&D System, USA) was used to determine the expression level of hTPO; HC-11 cell without transfection was used as a blank control and the empty vector pCDNA3.1 zeo(–) was used as a negative control.

### Generation of transgenic mice

Transgenic mice were generated using a method described previously^42^. All of the animal work and handling was carried out in accordance with the Guidelines of Animal Care and approved by Institutional Animal Care and Use Committee at Shanghai Institute of Medical Genetics (1999-2011). Five mammary gland-specific expression vectors were extracted by an Endofree Plasmid Purification kit (QIAGEN Inc., USA), dissolved in pyrogen-free water and digested with restriction enzymes Mun *I and* BSSH *II*. Target DNA fragments were purified by QIAquick Gel Extraction kit (QIAGEN Inc. USA) and S&S Elutip Minicolumns (QIAGEN Inc. USA), precipitated with ethanol, dissolved in DNA dilution buffer at a concentration of 2 ng/ul and centrifuged at 12,000rpm for 30min. Then the supernatants were microinjected into the pronuclei of fertilized C57BL/6 strain zygotes to generate transgenic mice following standard procedures.

### PCR detection for positive transgenic mice

TPO genome is highly conservative in human and mouse with approximately 90% homology. Therefore, human and mouse TPO cDNAs were compared to design specific primers, especially 3′ terminus of primers. Four primers (P1: 5′-TGCTTCTCCTAACTGCAAGGC-3′, P2: 5′-TGAGACAGATTCTGGGAGTG-3′, P3: 5′-ATGCCATCTTCCTGAGCTTC- 3′, P4: 5′-TTCAGAAGCCCAGAGCCAGTA- 3′) were synthesized. Primer P1/P4 and P2/P3 can amplify specific 620 bp and 610 bp fragments respectively. To screen transgenic mice, PCR was performed with tail genomic DNA for 30 cycles of 94 °C for 30 s, 64 °C for 1 min, and 72 °C for 1 min.

### ELISA for hTPO expression in the milk of transgenic mice

Female mice were separated from their pups for 3 hours after delivery, intraperitoneally injected prolactin (0.3IU) and then anesthetized with 0.25% tribromoethanol for 10 minutes. Next, mammary glands were gently massaged and milked with tweezers. According to the method described previously^43^,^44^, milk collection was slightly modified and started on day 8 after delivery. Briefly, samples were collected 3 times each day, 100 μl each time, and continually for 5 days. Then the milk was diluted 1:5 with pyrogen-free water and lipids were removed by centrifugation. 5 μL HCl (1N) were added per 200 μl milk, which was evenly mixed and centrifuged. The supernatants were tested for hTPO by Human TPO Immunoassay kit (R&D system, USA) following the instruction of manufacturer. The milk from normal mice served as a negative control.

### Western blot detection for hTPO expression in the milk of transgenic mice

The milk was diluted 1:2 with PBS, and equal volumes (20 μl) were loaded onto 10% SDS-PAGE gel under reducing conditions with the milk from normal mice as negative control. SDS-PAGE and following western blot were performed according to standard protocols. After blotting, the membrane was blocked for 1 h in TBS (pH 7.5) containing 0.2% Tween-20 and 10% BSA. As the first antibody, mouse anti-human TPO mAb (US biological) diluted 1:500 in 0.2% Tween-20 in TBS (pH 7.5) was incubated for 1 h at room temperature. The secondary antibody was HRP-conjugated goat anti-mouse IgG (H+L) (Jackson ImmunoResearch). The signal was developed using SuperSignal West Pico Chemiluminescent Substrate (Pierce).

### Detection for hTPO expression in the milk of lactating goat after the transfer of pTPOGD

Six lactating goats were bred separately. Three goats were set for experimental groups and another three goats for control groups. The milk of goat mammary gland was evacuated before injection. pTPOGD and pCDNA3.1 zeo(-) were purified by EndoFree Plasmid Maxi Purification Kit, embedded in liposome DOTAP and then slowly injected into the base of goat mammary with syringe. The milk was collected per 12 h after injection for continual 14 days and stored at −20 °C for hTPO measurement. The control group were injected with pCDNA3.1 zeo(-).

### Quantitative PCR for hTPO mRNA expression in HC-11 cells transfected with pTPOGA and pTPOGD

See Supplementary methods online.

### Construction of pTPO^△^GD

See Supplementary methods online.

### RT-PCR and quantitative RT-PCR for hTPO mRNA expression in HC-11 cells transfected with pTPOGD and pTPO^△^GD

See Supplementary methods online.

### Statistical Analysis

Data were representative of 3 independent experiments and expressed as mean  ±  standard deviation (SD). The results were processed using one-way ANOVA with the SPSS13.0 program. Significance was defined by p value of < 0.05.

## Additional Information

**How to cite this article**: Li, Y. *et al.* Intron V, not intron I of human thrombopoietin, improves expression in the milk of transgenic mice regulated by goat beta-casein promoter. *Sci. Rep.* **5**, 16051; doi: 10.1038/srep16051 (2015).

## Supplementary Material

Supplementary Information

## References

[b1] Gurney A. L. *et al.* Genomic structure, chromosomal localization, and conserved alternative splice forms of thrombopoietin. Blood 85, 981–988 (1995). 7849319

[b2] Chang M. S. *et al.* Cloning and characterization of the human megakaryocyte growth and development factor (MGDF) gene. J Biol Chem 270, 511–514 (1995). 7822271 10.1074/jbc.270.2.511

[b3] Kamura T., Handa H., Hamasaki N. & Kitajima S. Characterization of the human thrombopoietin gene promoter. A possible role of an Ets transcription factor, E4TF1/GABP. J Biol Chem 272, 11361–11368 (1997). 9111044 10.1074/jbc.272.17.11361

[b4] Ghilardi N., Wiestner A. & Skoda R. C. Thrombopoietin production is inhibited by a translational mechanism. Blood 92, 4023–4030 (1998). 9834204

[b5] Ghilardi N. & Skoda R. C. A single-base deletion in the thrombopoietin (TPO) gene causes familial essential thrombocythemia through a mechanism of more efficient translation of TPO mRNA. Blood 94, 1480–1482 (1999). 10484635

[b6] Buchman A. R. & Berg P. Comparison of intron-dependent and intron-independent gene expression. Mol Cell Biol 8, 4395–4405 (1988). 3185553 10.1128/mcb.8.10.4395-4405.1988 PMC365513

[b7] Moabbi A. M., Agarwal N., El Kaderi B. & Ansari A. Role for gene looping in intron-mediated enhancement of transcription. Proc Natl Acad Sci USA 109, 8505–8510, 1112400109 [pii]10.1073/pnas.1112400109 (2012). 22586116 10.1073/pnas.1112400109 PMC3365183

[b8] Callis J., Fromm M. & Walbot V. Introns increase gene expression in cultured maize cells. Genes Dev 1, 1183–1200 (1987). 2828168 10.1101/gad.1.10.1183

[b9] Duncker B. P., Davies P. L. & Walker V. K. Introns boost transgene expression in Drosophila melanogaster. Mol Gen Genet 254, 291–296 (1997). 9150263 10.1007/s004380050418

[b10] Palmiter R. D., Sandgren E. P., Avarbock M. R., Allen D. D. & Brinster R. L. Heterologous introns can enhance expression of transgenes in mice. Proc Natl Acad Sci USA 88, 478–482 (1991). 1988947 10.1073/pnas.88.2.478 PMC50834

[b11] Mascarenhas D., Mettler I. J., Pierce D. A. & Lowe H. W. Intron-mediated enhancement of heterologous gene expression in maize. Plant Mol Biol 15, 913–920 (1990). 2103480 10.1007/BF00039430

[b12] Parra G., Bradnam K., Rose A. B. & Korf I. Comparative and functional analysis of intron-mediated enhancement signals reveals conserved features among plants. Nucleic Acids Res 39, 5328–5337, gkr043 [pii]10.1093/nar/gkr043 (2011). 21427088 10.1093/nar/gkr043 PMC3141229

[b13] Ning Y. S. *et al.* [Role of intron and 5′ untranslated region in human thrombopoietin gene expression]. Di Yi Jun Yi Da Xue Xue Bao 24, 991–994 (2004). 15447843

[b14] Li Y., Zhou M., Zhou H. & Ning Y. The last intron of the human thrombopoietin gene enhances expression in milk of transgenic mice. Funct Integr Genomics 14, 229–236, 10.1007/s10142-013-0348-x (2014). 24287579

[b15] Kuter D. J. & Begley C. G. Recombinant human thrombopoietin: basic biology and evaluation of clinical studies. Blood 100, 3457–3469, 10.1182/blood.V100.10.3457100/10/3457[pii] (2002). 12411315

[b16] Kuter D. J. Whatever happened to thrombopoietin? Transfusion 42, 279–283 (2002). 11961230 10.1046/j.1537-2995.2002.00056.x

[b17] Kuter D. J. The biology of thrombopoietin and thrombopoietin receptor agonists. Int J Hematol 98, 10–23, 10.1007/s12185-013-1382-0 (2013). 23821332

[b18] Archibald A. L., McClenaghan M., Hornsey V., Simons J. P. & Clark A. J. High-level expression of biologically active human alpha 1-antitrypsin in the milk of transgenic mice. Proc Natl Acad Sci USA 87, 5178–5182 (1990). 1695012 10.1073/pnas.87.13.5178 PMC54285

[b19] Gordon K. *et al.* Production of human tissue plasminogen activator in transgenic mouse milk. 1987. Biotechnology 24, 425–428 (1992). 1422049

[b20] Velander W. H. *et al.* Production of biologically active human protein C in the milk of transgenic mice. Ann N Y Acad Sci 665, 391–403 (1992). 1416618 10.1111/j.1749-6632.1992.tb42602.x

[b21] Kosugi S. *et al.* Circulating thrombopoietin level in chronic immune thrombocytopenic purpura. British journal of haematology 93, 704–706 (1996). 8652398 10.1046/j.1365-2141.1996.d01-1702.x

[b22] Tahara T. *et al.* A sensitive sandwich ELISA for measuring thrombopoietin in human serum: serum thrombopoietin levels in healthy volunteers and in patients with haemopoietic disorders. British journal of haematology 93, 783–788 (1996). 8703803 10.1046/j.1365-2141.1996.d01-1741.x

[b23] Emmons R. V. *et al.* Human thrombopoietin levels are high when thrombocytopenia is due to megakaryocyte deficiency and low when due to increased platelet destruction. Blood 87, 4068–4071 (1996). 8639762

[b24] Ware J. Give me an intron: any intron. Blood 121, 4251–4252, 121/21/4251 [pii]10.1182/blood-2013-04-494856 (2013). 23704045 10.1182/blood-2013-04-494856

[b25] Lu S. & Cullen B. R. Analysis of the stimulatory effect of splicing on mRNA production and utilization in mammalian cells. Rna 9, 618–630 (2003). 12702820 10.1261/rna.5260303 PMC1370427

[b26] Nott A., Meislin S. H. & Moore M. J. A quantitative analysis of intron effects on mammalian gene expression. RNA 9, 607–617 (2003). 12702819 10.1261/rna.5250403 PMC1370426

[b27] Rose A. B. Intron-mediated regulation of gene expression. Curr Top Microbiol Immunol 326, 277–290 (2008). 18630758 10.1007/978-3-540-76776-3_15

[b28] Shabalina S. A. *et al.* Distinct patterns of expression and evolution of intronless and intron-containing mammalian genes. Mol Biol Evol 27, 1745–1749, msq086 [pii]10.1093/molbev/msq086 (2010). 20360214 10.1093/molbev/msq086 PMC2908711

[b29] Visel A., Rubin E. M. & Pennacchio L. A. Genomic views of distant-acting enhancers. Nature 461, 199–205, nature08451 [pii]10.1038/nature08451 (2009). 19741700 10.1038/nature08451 PMC2923221

[b30] Wang H. F., Feng L. & Niu D. K. Relationship between mRNA stability and intron presence. Biochemical and biophysical research communications 354, 203–208, 10.1016/j.bbrc.2006.12.184 (2007). 17207776 PMC7092898

[b31] Mor A. *et al.* Dynamics of single mRNP nucleocytoplasmic transport and export through the nuclear pore in living cells. Nature cell biology 12, 543–552, 10.1038/ncb2056 (2010). 20453848

[b32] Le Hir H., Nott A. & Moore M. J. How introns influence and enhance eukaryotic gene expression. Trends in biochemical sciences 28, 215–220, 10.1016/S0968-0004(03)00052-5 (2003). 12713906

[b33] Buhler T. A., Bruyere T., Went D. F., Stranzinger G. & Burki K. Rabbit beta-casein promoter directs secretion of human interleukin-2 into the milk of transgenic rabbits. Biotechnology (NY) 8, 140–143 (1990). 10.1038/nbt0290-140 1366359

[b34] Uusi-Oukari M. *et al.* Bovine alpha s1-casein gene sequences direct high level expression of human granulocyte-macrophage colony-stimulating factor in the milk of transgenic mice. Transgenic research 6, 75–84 (1997). 9032980 10.1023/a:1018461201385

[b35] Brinster R. L., Allen J. M., Behringer R. R., Gelinas R. E. & Palmiter R. D. Introns increase transcriptional efficiency in transgenic mice. Proc Natl Acad Sci USA 85, 836–840 (1988). 3422466 10.1073/pnas.85.3.836 PMC279650

[b36] Garrick D., Fiering S., Martin D. I. & Whitelaw E. Repeat-induced gene silencing in mammals. Nature genetics 18, 56–59, 10.1038/ng0198-56 (1998). 9425901

[b37] Burkov I. A. *et al.* Expression of the human granulocyte-macrophage colony stimulating factor (hGM-CSF) gene under control of the 5′-regulatory sequence of the goat alpha-S1-casein gene with and without a MAR element in transgenic mice. Transgenic research 22, 949–964, 10.1007/s11248-013-9697-4 (2013). 23435752

[b38] Li H. *et al.* Expression of biologically active human interferon alpha 2b in the milk of transgenic mice. Transgenic research 22, 169–178, 10.1007/s11248-012-9623-1 (2013). 22661167

[b39] Qian X., Kraft J., Ni Y. & Zhao F. Q. Production of recombinant human proinsulin in the milk of transgenic mice. Scientific reports 4, 6465, 10.1038/srep06465 (2014). 25267062 PMC4179469

[b40] Vadhan-Raj S. *et al.* Safety and efficacy of transfusions of autologous cryopreserved platelets derived from recombinant human thrombopoietin to support chemotherapy-associated severe thrombocytopenia: a randomised cross-over study. Lancet 359, 2145–2152, 10.1016/S0140-6736(02)09090-6 (2002). 12090979

[b41] Lubo H. & Palmer C. Transgenic animal bioreactors–where we are. Transgenic research 9, 301–304 (2000). 11131008 10.1023/a:1008989619924

[b42] Min W. *et al.* 307-bp fragment in HOXA7 upstream sequence is sufficient for anterior boundary formation. DNA Cell Biol 17, 293–299 (1998). 9539109 10.1089/dna.1998.17.293

[b43] Bagis H. *et al.* Expression of biologically active human interferon gamma in the milk of transgenic mice under the control of the murine whey acidic protein gene promoter. Biochemical genetics 49, 251–257, 10.1007/s10528-010-9403-7 (2011). 21170579

[b44] Zhou Y. *et al.* The high-level expression of human tissue plasminogen activator in the milk of transgenic mice with hybrid gene locus strategy. Molecular biotechnology 50, 137–144, 10.1007/s12033-011-9428-0 (2012). 21688038

