# Supplementary material for: Intron V, not intron I of human thrombopoietin, improves expression in the milk of transgenic mice regulated by goat beta-casein promoter
Source: Sci Rep. 2015 Nov 3;5:16051. doi: 10.1038/srep16051 (PMC4630608; doi:10.1038/srep16051)
Supplement: Supplementary Information [file srep16051-s1.doc]

**Supplementary information**

**Intron Ⅴ, not intronⅠof human thrombopoietin, improves expression in the milk of transgenic mice regulated by goat beta-casein promoter**

Yan Li^1#^, Hu Hao ^2#^, Mingqian Zhou^1^，Hongwei Zhou^3^, Jianbin Ye^1^，Lijun Ning^1^, Yunshan Ning1*

**Supplementary methods**

**Construction of TPO mammary gland specific expression vectors**

The schematic depictions of targeting constructs including pTPOGA, pTPOGB, pTPOGC, pTPOGD and pTPOGE were shown in Fig.1 and the corresponding vectors in construction were referred to this publication[^13^](#_ENREF_13). pCβ-casein (containing 6.5 kb **β-**casein promoter) and pCTPO (containing TPOcDNA/ pCDNA3.1 ) vectors were linear by Not *I* respectively. The gaps were subsequently filled with *Klenow* polymerase and 6.5 kb **β**-casein and 6.0 kb TPOcDNA/pCDNA3.1 fragments were recovered, ligated and transformed. Recombinant **β**-casein- TPOcDNA / pCDNA3.1 vector was screened and named as pTPOGA. The pBβ-casein and pT1-2TPO (TUR) vectors were digested by Not*I* and *BamH* I respectively. 6.5kb β-casein promoter fragment and 2.0kb TUR fragment were recovered, ligated and transformed. Recombinant β-casein- TUR-TPOcDNA / pCDNA3.1 vector was screened and named as pTPOGB. The vector pTPOGB and pCDT1-2TPO (ΔTUR) were digested by *BamH* I and TUR was replaced by ΔTUR. The recombinant β-casein-ΔTUR-TPO cDNA/pCDNA3.1 zeo(一) vector screened and named pTPOGC. The vector pCT7-8 TPO (intronⅤ-TPOcDNA) and pTPOGA were digested by *BamH* I. 1.3 kb intron Ⅴ-TPO cDNA and 11.5 kb pTPOGA were recovered, ligated and transformed. The recombinant β-casein -TPO intron V-TPO cDNA/pCDNA3.1 zeo(-) were constructed and named as pTPOGD. The pBβ-casein and pCTPOg(TPO genomic DNA) vectors were digested by Not*I,* 6.5kb β-casein promoter fragment and 6.2 kb TPO genomic DNA fragment were recovered, ligated and transformed. The recombinant β-casein TPO genomic DNA/pCDNA3.1 zeo(-) were constructed and named as pTPOGE. All constructs were confirmed by restriction enzyme analysis and sequencing for both strands of the insert and flanking regions.

**Quantitative RT-PCR for hTPO mRNA expression in HC-11 cells transfected by pTPOGA and pTPOGD**

HC-11 cells were transiently transfected with pTPOGA and pTPOGD for 48h by DOTAP liposome, respectively. Total cellular RNA was extracted from transfected cells using Trizol (Invitrogen) and then was reverse-transcribed using PrimeScript RT reagent Kit With gDNA Eraser (TaKaRa, Tokyo, Japan). Real-time quantitative RT-PCR was performed in triplicate using the specific primers and the TaqMan probe (shown in Supplementary Table S2) in an ABI PRISM Stepone Plus Sequence Detection System (Applied Biosystems 7500, Foster City, CA, United States). PCR protocol was: 95°C for 10 minutes followed by 50 cycles of 95°C for 15 seconds and 61°C for 1 minute. β-actin was used as internal control. Relative quantitation of gene expression was detected using the delta delta Ct (threshold cycle) method. Briefly, the averages and standard deviations were determined for triplicates. ∆Ct is the difference between Ct of target mRNA and Ct of β-actin for each group.

**Construction of pTPO**^△^**GD**

With pCT7-8 TPO as template, ^△^intron Ⅴ (5' splice site of intron Ⅴwas mutated from GT to CA) was amplified using specific primers （sense 5'-CCCTGCAGAGCCTCCTTGGAACCCAGCA-3', antisense5'-CAGGAAGATGGCATTGGGATCCTT -3'） .Then the PCR product and pCT7-8 TPO were digested by Pst*Ⅰ* and BamH *Ⅰ*. 0.3 kb intron Ⅴ and 5.9 kb pCTPO were recovered, ligated and transformed to construct recombinant p^△^CT7-8TPO. After that, the vector p^△^CT7-8 TPO and pTPOGA were digested by BamH *Ⅰ*. 1.3 kb ^△^intron Ⅴ-TPO cDNA and 11.5 kb pTPOGA were recovered, ligated and transformed to construct recombinant pTPO^△^GD. All constructs were confirmed by restriction enzyme analysis and sequencing for both strands of the insert and flanking regions.

**RT-PCR and quantitative RT-PCR for hTPO mRNA expression in HC-11 cells transfected with pTPOGD and pTPO^△^GD**

pTPOGD and pTPO△GD were respectively transfected into HC-11 cells for 48h and total cellular RNA was extracted from cells using the method described above. Nascent RNA was amplified using RT-PCR/qRT-PCR. RT-PCR was carried out in two steps. In the first step 2 μg of RNA was reverse transcribed into cDNA using oligo(dT) primer and SuperScript III reverse transcriptase (Invitrogen) according to the manufacturer's instructions. In the second step, poly(A) mRNA derived cDNA was PCR-amplified by specific primers (sense prime: 5’-AGACCAAGGCACAGGACATTC-3’, antisense prime: 5’-CACAGGGGTGGGCAAGGT-3’. The PCR profile was as follows: 94 °C 2 min, 35 cycles of 94 °C 30 s, 62 °C 30 s, and 72°C 1 min, and 72°C 10 min. Quantitative RT-PCR was performed as described in Methods above.

**Supplementary Tables**

**Table S1 Detection** of the hTPO expression in the milk of transgenic mice

| **Numbers** | **Gene construction** | **Concentration (ng/ml)** |
| --- | --- | --- |
| #10 | β-casein -△TUR-TPO cDNA | 2.00±0.48 |
| #46 | β-casein -△TUR-TPO cDNA | 1.1±0.63 |
| #38 | β-casein –intronⅤ-TPO cDNA | 146.00±26.55 |
| #50 | β-casein - TUR-TPO cDNA | 1.22±0.42 |
| #49 | β-casein –intronⅤ-TPO cDNA | 122.80±38.49 |
| #42 | β-casein -TPOcDNA | 8.10±2.17 |
| #16 | β-casein -TPOgDNA | 0.49±0.11 |

TPO expression levels were obtained with an ELISA following manufacturer’s protocol. Expression levels represented the value of milk pooled from 7 female mice at day 8 of lactation for each F0 transgenic line. The data represented the mean±standard deviation for 3 independent experiments for each transgenic mice.

**Table S2** Primers and probes used in real time quantitative PCR

| **Names** | **Primers and probe** |
| --- | --- |
| hTPO | Forward: 5’- CCAAGATTCCTGGTCTGCTGA-3’  Reverse: 5’- CACAGGGGTGGGCAAGGT-3’  Probe: 5’-CTGAACAGGATACACGAACTCTTGAATGGA-3’ |
| β-actin | Forward: 5’-GGAAATCGTGCGTGACATTAAG-3’ |
|  | Reverse: 5’-AGGAGCTGGAAGCAGCC-3’ |
|  | Probe: 5’-CTACGTCGCCCTGGACTTCG-3’ |

**Supplementary Figures**

**
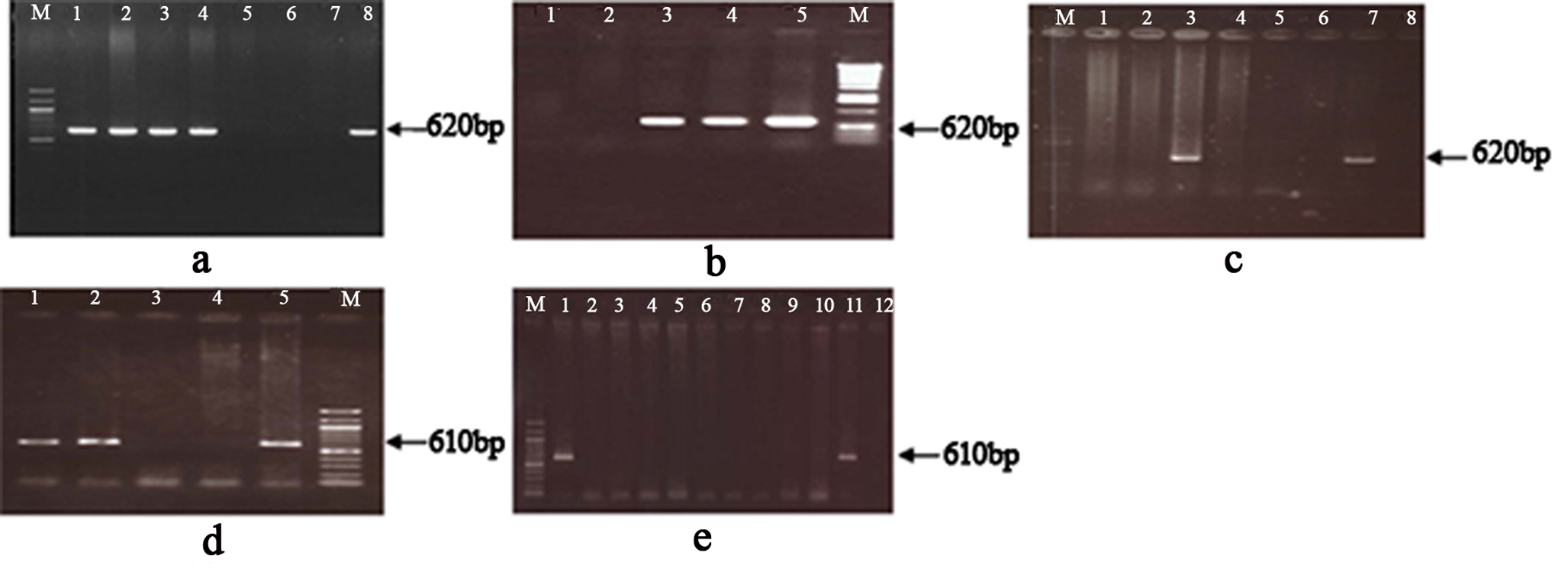
**

Figure S1 Identification of transgenic mice carrying the corresponding vectors by PCR analysis. PCR analysis of the tail DNA from transgenic mice was performed with the primer pairs. PCR products were generated from positive transgenic mice after 30 cycles (94°C for 30s, 64°C for 1 min, 72℃ for 1 min). Tail DNA from a normal mouse was used as negative control. (a) Identification of transgenic mice carrying β-casein-TPOcDNA fusion gene. M: 100bp DNA ladder; 1-6, 8: transgenic mice; 7: normal mice. (b) Identification of transgenic mice carrying β-casein -TUR-TPO cDNA fusion gene. M: 100bp DNA ladder; 1: normal mice; 2-5: transgenic mice. (c) Identification of transgenic mice carrying β-casein -△TUR-TPO cDNA fusion gene. M: 100bp DNA ladder; 1: normal mice; 2-8: transgenic mice. (d) Identification of transgenic mice carrying β-casein- intronV- TPO cDNA fusion gene. M: 100bp DNA ladder; 1-3, 5: transgenic mice; 4: normal mice. (e) Identification of transgenic mice carrying β-casein -TPOgDNA fusion gene. M: 100bp DNA ladder; 1-11: transgenic mice; 12: normal mice.

**
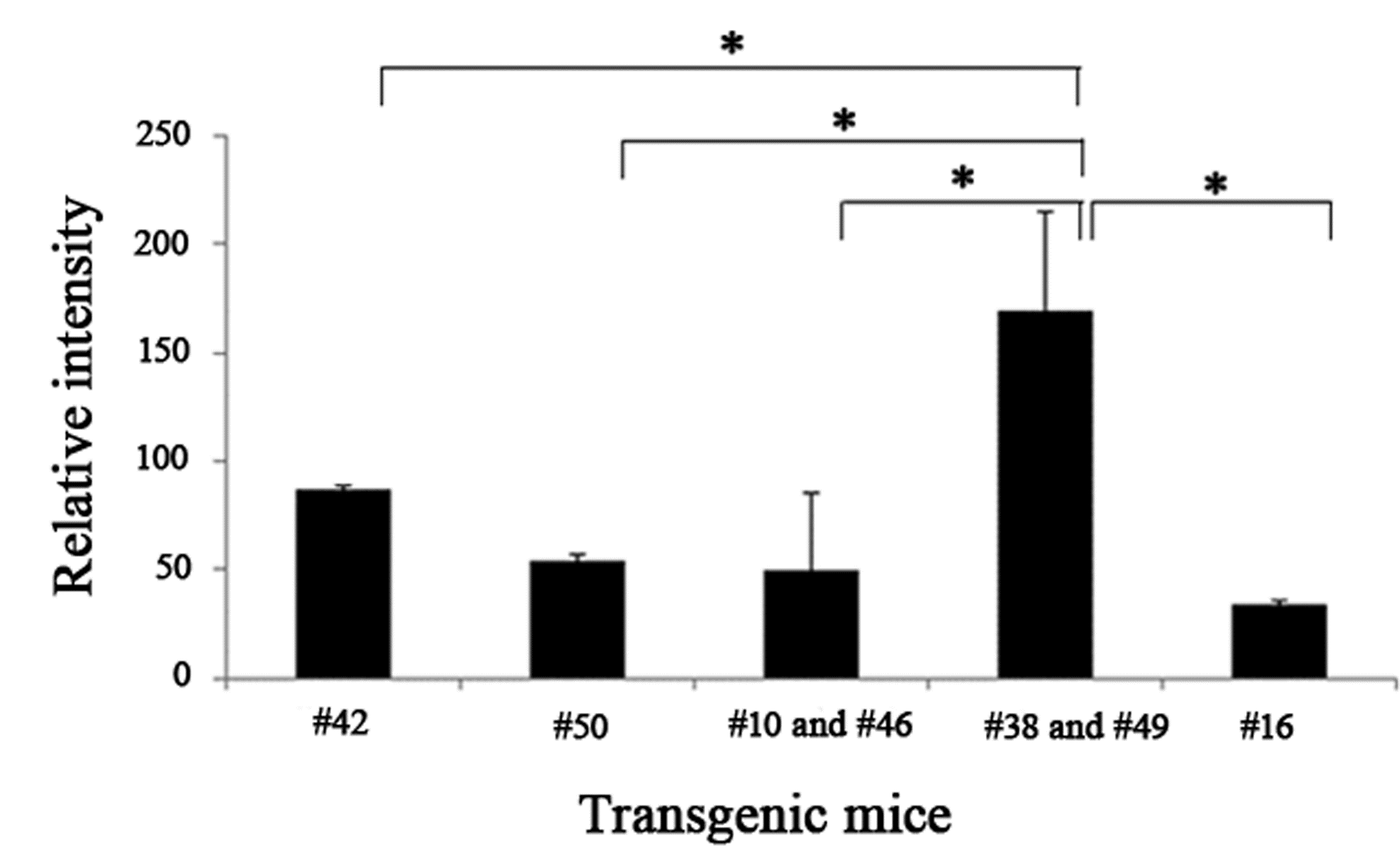
**

Figure S2 Statistical analysis of hTPO expression in the milk of transgenic mice. After hTPO expression in the milk of transgenic mice was determined by western blot, the band relative intensity of hTPO expression was quantified by Gel-Pro Analyzer 4.0 software. Then statistical analysis was performed by one-way ANOVA using SPSS13.0 program. Significance was defined by p value of < 0.05.*p <0.05.


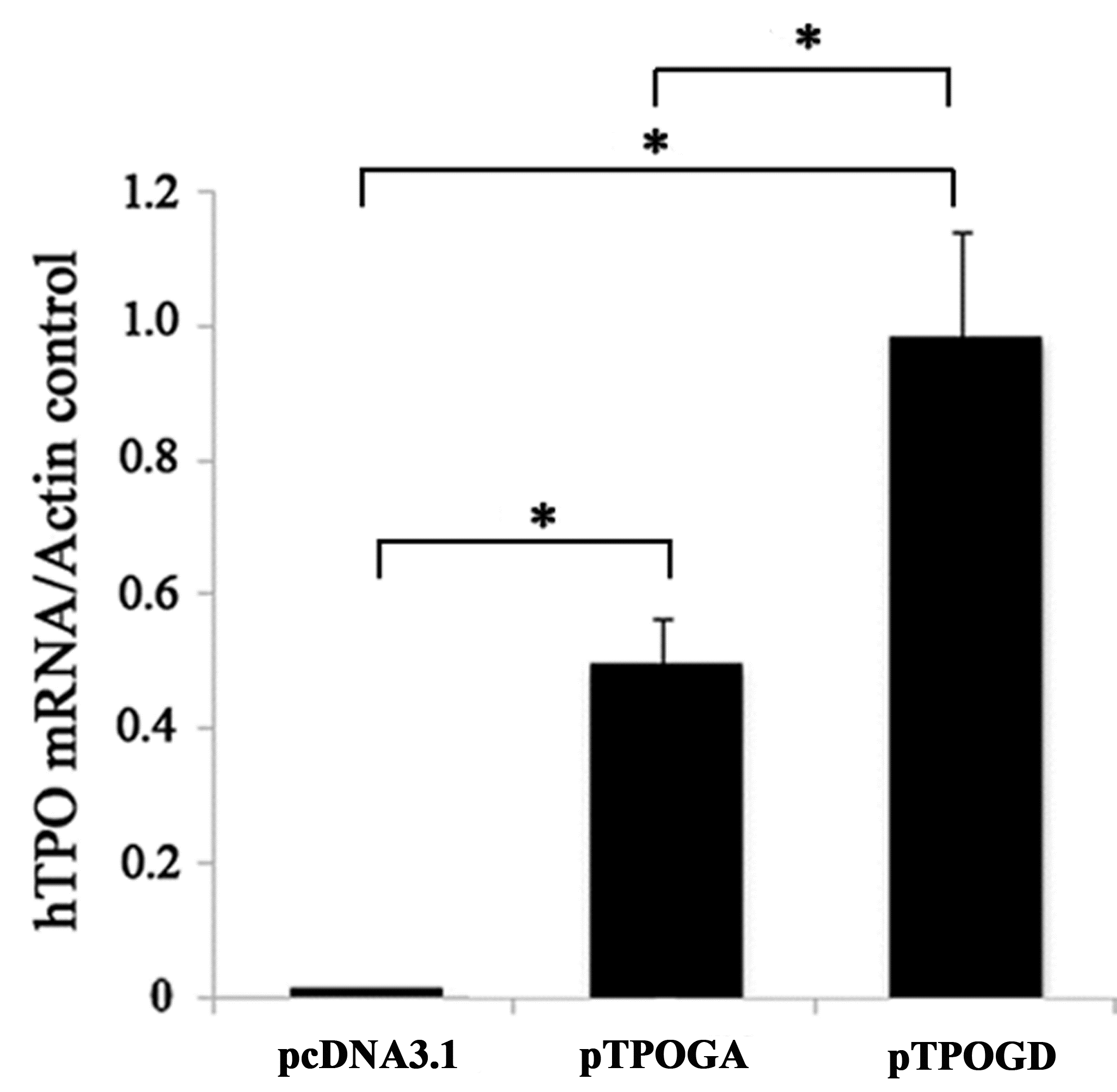


Figure S3 Relative expres­sion of hTPO mRNA in HC-11 cells transfected with pTPOGA and pTPOGD. Quantitative RT-PCR was performed to compare hTPO mRNA expression in HC-11 cells transfected with pTPOGA and pTPOGD expression vectors, respectively. The experiments were carried out in triplicate and data was expressed as mean ± S.D. p<0.05 was considered as statistically significant *p<0.05.

**
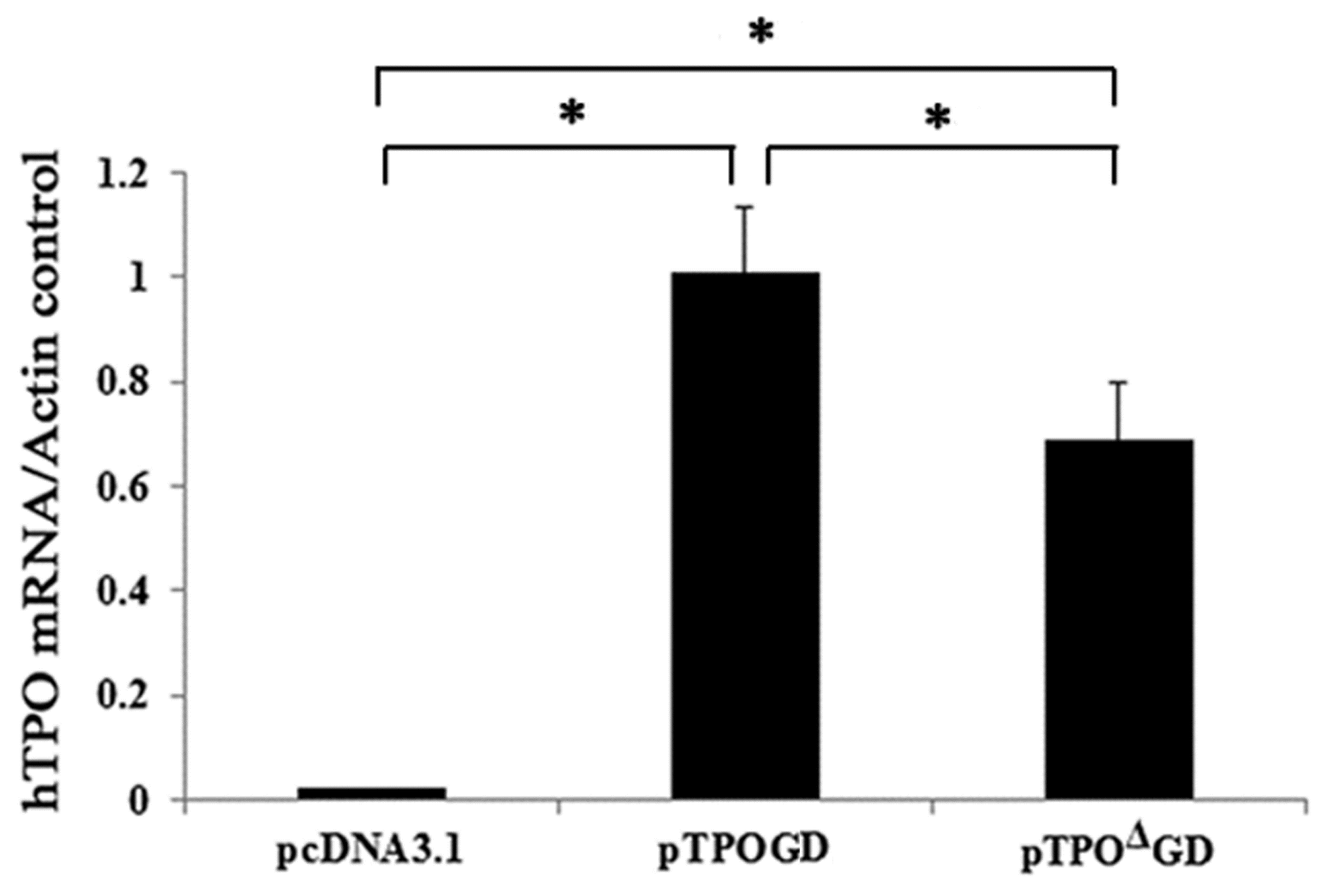
**

Figure S4 Relative expres­sion of hTPO mRNA in HC-11 cells transfected with pTPOGD and pTPO^Δ^GD. HC-11 cells was transfected with pTPOGD and pTPO^Δ^GD expression vectors, respectively. Quantitative RT-PCR was performed to compare hTPO mRNA expression in cells. The experiments were carried out in triplicate and data was expressed as mean ± S.D. p<0.05 was considered as statistically significant. * P<0.05.
